# The Stability of Synthetic Cathinones and the Study of Potential Intake Biomarkers in the Biological Material from a Case of 3-CMC Poisoning

**DOI:** 10.1093/jat/bkad010

**Published:** 2023-02-15

**Authors:** Agnieszka Romańczuk, Sebastian Rojek, Kamil Synowiec, Martyna Maciów-Głąb, Karol Kula, Ewa Rzepecka-Woźniak

**Affiliations:** Department of Forensic Medicine, Faculty of Medicine, Jagiellonian University Medical College, Grzegórzecka 16 St., Kraków 31-531, Poland; Department of Forensic Medicine, Faculty of Medicine, Jagiellonian University Medical College, Grzegórzecka 16 St., Kraków 31-531, Poland; Department of Forensic Medicine, Faculty of Medicine, Jagiellonian University Medical College, Grzegórzecka 16 St., Kraków 31-531, Poland; Department of Forensic Medicine, Faculty of Medicine, Jagiellonian University Medical College, Grzegórzecka 16 St., Kraków 31-531, Poland; Department of Forensic Medicine, Faculty of Medicine, Jagiellonian University Medical College, Grzegórzecka 16 St., Kraków 31-531, Poland; Department of Forensic Medicine, Faculty of Medicine, Jagiellonian University Medical College, Grzegórzecka 16 St., Kraków 31-531, Poland

## Abstract

The objective of the present study was to identify the metabolites of synthetic cathinone (SC), 3-chloromethcathinone (3-CMC), and to select a potential intake biomarker for this compound. The basis of the experiment was the analysis of blood and urine samples from a case of fatal poisoning with this substance. We also evaluated the stability of 3-CMC and the selected potential biomarker, the dihydro-3-CMC metabolite, depending on the time elapsed since the autopsy as well as the storage conditions of the biological material. Studies have shown that 3-CMC shows low stability in the biological material. However, acidification of the biological material or storage at low temperatures has a great impact on maintaining its stability. It is crucial to monitor the presence of the dihydro-3-CMC metabolite in the biological material during analysis, which showed high stability under all of the given storage conditions. SCs might not be detected in the biological material and, if they are, the determined concentrations at the time of testing may not correspond to the actual concentrations at the time of the event or death. Consequently, the interpretation of the results obtained for 3-CMC and dihydro-3-CMC in terms of assessing their toxicity and possible cause of death is difficult. The area of research into the search for other intake biomarkers of unstable halogen derivatives of SCs remains open.

## Introduction

New psychoactive substances (NPSs) are constantly popular among drug users. Each year, dozens of NPSs appear in the European Union’s Early Warning System (EWS) of the European Monitoring Center for Drugs and Drug Addiction (EMCDDA). By the end of 2021, the EMCDDA was already monitoring ∼880 NPSs, 52 of which were reported in Europe for the first time in 2021 (1). NPSs are currently one of the most serious toxicological threats, affecting many areas of society, including legislation, forensic medicine and public health, but they are also a significant clinical problem.

NPSs are a complex and diverse group of substances. They are analogs of existing controlled drugs and pharmaceutical products, which mimic their effects through similar interactions with the central nervous system (2). Considering the psychoactive effects they produce, NPSs can be divided into six groups: opioids (e.g., furanylfentanyl), cannabimimetics (e.g., AMB-FUBINACA), dissociatives (e.g., deschloroketamine), hallucinogens (e.g., 1p-LSD), sedatives (e.g., etizolam) and stimulants (e.g., dimethylcathinone) (3).

While many NPSs often disappear from the drug market rather quickly, some are still found due to their high popularity among users. Among these substances, synthetic cathinones (SCs) are one of the most widespread classes of NPSs that have become popular due to their psychostimulant and hallucinogenic effects being similar to cocaine, 3,4-methylenedioxymethamphetamine (MDMA) and amphetamines (4). SCs also represent the second largest category of NPSs monitored by the European Union’s EWS. At the end of 2021, the EMCDDA was monitoring 162 SCs (1).

The high structural variability of SCs, combined with the absence of knowledge of their metabolic transformations, creates a great challenge for toxicological analysis. An important problem, from the point of view of toxicological poisoning evaluation, is also their stability in the biological material. Understanding the stability of analyzed xenobiotics in biological samples is crucial for the proper interpretation of the analytical results. Loss of analytes can occur due to chemical degradation, enzymatic metabolism or the presence of interfering substances due to degradation of the biological matrix, including (but not limited to) inappropriate sample storage, handling and/or transport conditions (5). In view of the low stability of SCs, it may be crucial to search for potential biomarkers of admission that can demonstrate greater stability in the biological material.

3-Chloromethcathinone (3-CMC, 1-(3-chlorophenyl)-2-(methylamino)propan-1-one) was for the first time identified on the European drug market in September 2014, following a police seizure in Sweden (6). In addition, the first mentions on Polish internet forums, posted by NPS users, about taking 3-CMC appeared in April 2014 (7). The substance is a halogenated (in the aromatic ring) and *N*-alkylated derivative of cathinone. It has a center of chirality, so there can be two enantiomers: (*R*)-3-CMC and (*S*)-3-CMC. 3-CMC is similar in both structure and induced stimulant effects to methcathinone (ephedrone) and 4-chloromethcathinone (4-CMC, clephedrone). 3-CMC has two constitutional isomers: 2-chloromethcathinone (2-CMC) and 4-CMC. Distinguishing 3-CMC from 2- and 4-CMC requires appropriate analytical techniques (6).

The purpose of the present study was to identify the metabolites of SC, 3-CMC, and to select a potential biomarker for the intake of this substance based on the analysis of blood and urine samples from a case of fatal poisoning with this substance. We also evaluated the stability of 3-CMC and its potential biomarker, the dihydro-3-CMC metabolite, depending on the elapsed time since the autopsy as well as the storage conditions of the biological material. The experiment was based on an untypical case of a man, in which circumstances of finding a corpse allowed the detection of 3-CMC and the appropriate preservation of biological material for the stability experiment on the same day of the autopsy.

## Case History

A 42-year-old male was found with no signs of life on the floor in a church apartment. The body was in a lying position and was naked. The man had only a pair of wireless headphones on his ears. In the area of his left ankle, he had a peripheral intravenous cannula (Venflon) inserted and taped in place with a plaster that was unrelated to any medical procedures. Numerous bruises and injection marks were visible on both lower extremities. Several pieces of physical evidence were revealed next to the body and in the room, including empty peripheral intravenous cannula packages, used cannulas, paper towels contaminated with a red-colored substance, intimate gel packages, empty syringes and needles. In addition, two ampules of “Biostimine 1 mL do not inject” and a glass jar filled with a white crystalline powder with a sticker reading “Vit C sodium ascorbate” were revealed.

The medicolegal autopsy was performed at the Department of Forensic Medicine in Kraków 2 days after the discovery of the body. No additional external changes were found during the inspection of the body. The internal examination revealed no injuries related to death as a result of mechanical trauma or pathological changes that would unequivocally explain the cause of death. Pulmonary edema, hyperemia of internal organs, enlargement of the heart cavities, slight atherosclerosis of the coronary arteries, signs of hepatic steatosis and scars in the kidneys cortex were reported, which were further confirmed by a microscopic examination. Moreover, a histopathological examination showed fine myocardial fibrosis and diffuse necrosis of single hepatocytes; subcutaneous tissue specimens from the area of the lesions of the left lower leg presented chronic reactive (inflammatory) changes relevant to chronic injections.

Samples of blood and urine were collected during the autopsy for a toxicological examination, including ethyl alcohol evaluation. The peripheral intravenous cannula was also secured.

A further search of the decedent’s apartment revealed additional 106 pieces of fragments of string bags with traces of white powder, which were also shipped for toxicological testing.

## Materials and Methods

### Biological and non-biological materials

- Blood and urine samples were collected during the autopsy of the deceased.
- Blank whole blood samples used for the development and validation of the analytical method were purchased from the Regional Blood Donation and Blood Treatment Center in Kraków (*n* = 3).
- Fluid from the Venflon was secured during the autopsy of the deceased.

### Standards and chemicals

The standard solution of 3-CMC was purchased from Cayman Chemical (USA) and 4-chloroethcathinone-d_5_ (4-CEC-d_5_), used as an internal standard (IS), was from Cerilliant (USA).

Bond Elut solid-phase extraction (SPE) columns filled with a 500-mg bed of non-polar silica gel modified with the octadecyl (C18-EC) phase were sourced from Agilent Technologies (USA). The acetonitrile, acetic acid, ammonium formate, formic acid, methanol and acetonitrile for high-performance liquid chromatography coupled to mass spectrometry were obtained from Merck (Poland).

## Identification Analysis of the Fluid from the Venflon and Urine Sample

The fluid from the Venflon was acetylated and then analyzed by gas chromatography coupled to mass spectrometry (GC--MS), as described by Synowiec et al. (8). Screening analysis was performed using a library developed by Pfleger, Maurer and Weber (9), which contains a broad group of compounds, including acetyl or trimethylsilyl derivatives, as well as the authors’ library of SCs (8). The analysis was conducted on the day of the autopsy, which was 2 days after the body was discovered.

Following preliminary analytical results indicating the presence of chloromethcathinone (CMC), the liquid from the Venflon further underwent a trimethylsilylation process (8) to distinguish the constitutional isomer of CMC.

The same procedure was carried out in the urine sample after SPE, according to the methods described in the authors’ earlier works (10, 11).

## Preservation of Biological Material

**Table I.** Fragmentation Parameters for Analyte and IS

Compound name	Precursor ion	Product ion	Fragmentor voltage [V]	Collision energy [V]	Retention time [min]
3-CMC	198.2	**180.1**	65	9	4.7
		144.1		33	
4-CEC-d_5_	217.2	**164.1**	71	17	4.9
		199.1		9	

The bold values indicate the quantitative ions.

Due to the positive result of the identification analysis of the fluid from the Venflon, the biological material (blood and urine) was properly preserved on the same day. In the case of the urine sample, the pH was first measured using a universal indicator paper (pH = 6.0). The blood sample was preserved in a tube with sodium fluoride (NaF) and potassium oxalate (KOx). The biological material was divided into three parts, each with a volume of 1 mL. The first part was stored at 4°C. The second part was acidified with 1 M HCl: 100 µL of HCl was added to 1 mL of the blood sample to obtain pH <7, and 50 µL of HCl was added to 1 mL of the urine sample to obtain pH = 4.2. The third part of the blood and urine samples was stored at −30°C.

## Screening Tests

Screening tests included the analysis of the man’s blood samples using enzyme-linked immunosorbent assay (ELISA) kits from Neogen (UK) for the presence of opiates, cocaine, amphetamine and its derivatives, methamphetamine/MDMA, cannabinoids, benzodiazepines, barbiturates and tricyclic antidepressants.

In addition, the blood and urine samples were analyzed for the presence of alkaline, neutral and acidic pharmacological agents using the high performance liquid chromatography with photodiode array detection method in the Merck Tox Screening System developed by Merck (Germany).

## Analysis of Biological Material

The 200 µL of blank, calibration and quality control (QC) blood samples and the man’s blood and urine samples were enriched with the addition of methanolic IS 4-CEC-d_5_. For the blood sample, the final IS concentration was 100 ng/mL, and for the urine sample, it was 1,000 ng/mL. A homogenizing element in the form of a 5-mm-diameter stainless steel bead was added to the Eppendorf Tubes containing the samples. The samples were then deproteinized with frozen acetonitrile (400 µL). The samples were then centrifuged, and the supernatant was evaporated in a stream of nitrogen in a 40°C sand bath. The dry residue was dissolved in 100 µL of a mixture of phase A and B solution in a volume ratio of 60:40 (*v/v*). Mobile phase A is a mixture of 2 mM ammonium formate and 0.2% formic acid solution in water (*v/v*), and phase B is a mixture of 2 mM ammonium formate and 0.2% formic acid solution in acetonitrile (*v/v*).

## High-Performance Liquid Chromatography--Electrospray Ionization Tandem Mass Spectrometry (HPLC--ESI-MS-MS) Method

An Agilent 1200 liquid chromatograph (Agilent) equipped with a binary pump (G1312 A) and an autosampler (G1329 A) was used. The chromatographic separation was performed with a Poroshell 120 EC-C18 column (3.0 × 100 mm, particle size 2.7 μm, Agilent). The column was thermostated at 40°C.

The flow of the mobile phases through the chromatographic column occurred at a programmed gradient of mobile phase composition and flow rate (initially, 95% of phase A and 5% of phase B at a flow rate of 0.5 mL/min; then, the proportion of phases was increased linearly to 90% of phase B at a flow rate of 1.0 mL/min at 10 minutes, and the condition was maintained for 2 min). Subsequently, the proportion of phases was increased linearly to 95% of phase B at a flow rate of 1.0 mL/min at 15 minutes. The volume of the samples injected into the chromatographic column was 15 μL.

A 6410 triple-quadrupole mass spectrometer (Agilent) with an electrospray ionization (ESI) source, operated under a positive mode, was used. The operational parameters of the ESI source were as follows: vaporizing temperature 350˚C, pressure of the nebulizing gas 40 psi, flow of the drying gas 9 L/min and capillary potential 3.5 kV. The multiple reaction monitoring (MRM) was employed. The fragmentation parameters of the analyzed compounds are listed in Table I. For the analysis of the selected potential biomarker, dihydro-3-CMC, the total ion current scanning mode was used in the *m*/*z* range of 50–650 amu.

## Process Validation

To assess the selectivity of the method, blank blood samples (*n* = 3) without added analyte and IS were subjected to extraction and analysis. Potential interferences from the biological matrix were evaluated in the 3-CMC or IS elution areas.

The specificity of the method was assessed by analyzing a mixture of 78 compounds, including SCs, amphetamine and its derivatives, at a concentration of 1,000 ng/mL each.

The calibration curve was generated based on the analysis of blank blood samples containing a known amount of 3-CMC. The blood samples were spiked with 3-CMC at the concentrations of 50, 100, 250, 500, 1,000 and 2,500 ng/mL. Each calibration sample was prepared in triplicate. The calibration samples were deproteinized with frozen acetonitrile according to the previously described procedure.

Intra-day and inter-day accuracy and precision were evaluated for two 3-CMC concentrations: 125 ng/mL (QC1) and 1,250 ng/mL (QC2). Intra-day accuracy and precision were determined for one batch, in three replicates, for QC1 and QC2. Inter-day accuracy and precision were determined for three separate batches, in three replicates, for QC1 and QC2. The accuracy of the method was presented as relative error (%). The precision of the method was expressed as the relative standard deviation (% RSD).

The limit of detection (LOD) of the method was determined as the lowest concentration at which the signal-to-noise (S/N) ratio, calculated as the height of the peak obtained for the defined MRM pair with a lower intensity (198.2→180.1), was ≥10. The limit of quantification (LOQ) of the method was adopted as the lowest concentrations for which the LOD criteria were fulfilled; the accuracy of the method was in the range of 80–120% of the actual concentration, and the precision of the method was ≤20% of RSD.

## Stability Study

While planning the experiment, the limitation was the amount of the biological material secured during the autopsy, as it came from an authentic case. The reference point (day 1) was the extraction of the biological material stored at 4°C for 24 hours using the procedure described earlier. The deproteination was performed the day after the autopsy was conducted, and the biological material was preserved. Subsequently, all the adequately preserved biological material was examined similarly after 2, 4 and 12 months. These intervals were chosen on the basis of the time that elapses between the autopsy and the intervals on which toxicological examinations are most often ordered to the Toxicology Laboratory of the Department of Forensic Medicine in Krakow by the Prosecutor’s Office. A period of 12 months is an extreme interval, but it also does occur in the Toxicology Laboratory. In addition to the parent substance, the metabolite dihydro-3-CMC, selected on the basis of two complementary methods (GC--MS and HPLC--ESI-MS), was also analyzed during stability studies in order to assess its usefulness as a potential intake biomarker of 3-CMC.

## Results and Discussion

To study the selectivity and specificity of the method for the determination of 3-CMC in blood samples, no significant interferences were found in the analyte elution or IS areas. However, because the constitutional isomers of CMC have identical mass spectra and retention times, they will therefore result in the same analytical signal. Complementary methods are needed to differentiate them. The parameters of the calibration curve and LOD and LOQ are shown in Table II. The accuracy and precision results for 3-CMC were within the ranges of −19.9 to 7.3 and 7–10% RSD, respectively.

**Table II.** Validation Parameters of the Developed Method for the Determination of 3-CMC in Blood Samples

Compound name	Calibration curve (*n* = 3)	Coefficient of determination (*R*^2^)	LOD [ng/mL]	LOQ [ng/mL]
3-CMC	*y *= −0.0008*x*^2^ + 0.3190*x *+ 0.0072	0.9919	10	50

The blood samples collected during the autopsy of the deceased did not show the presence of ethyl alcohol. The results obtained from the screening of the man’s blood samples using ELISA tests were positive for compounds of the amphetamine and methamphetamine/MDMA groups.

The unusual circumstances in revealing the body and the presence of a Venflon in the deceased, which had not been attached during the medical procedures performed, prompted the authors to analyze the fluid from the Venflon immediately after the autopsy. The preliminary analysis using the GC--MS method revealed the presence of one of the constitutional isomers of CMC. Following literature reports and the authors’ own experience regarding its stability, an experiment was planned to identify the metabolites, to select a potential intake biomarker of this substance and to examine the stability of CMC, along with its biomarker, in authentic biological material.

Acetylation of the fluid from the Venflon was performed for screening analysis. Then, after a positive analysis for CMC, a trimethylsilylation process was performed to discriminate the constitutional isomer of CMC. An analogous procedure was carried out in the man’s urine sample. Acetyl derivatives of the constitutional isomers of CMC do not allow for their identification, especially in the case of biological material, since the retention times of the individual CMC isomers are very similar. However, after the acetylation process, we obtain chromatograms that are more transparent and easier to interpret but most importantly can be compared with the published literature data of SCs, as well as their metabolites. In the case of trimethylsilyl derivatives, it is possible to separate the constitutional isomers of CMC.

**Figure 1.**
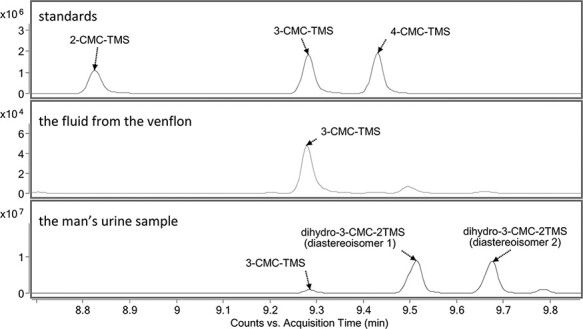
Comparative analysis of CMC constitutional isomer standards, the fluid from the Venflon and the man’s urine sample after performing trimethylsilylation.

The analysis result of the fluid from the Venflon after the trimethylsilylation process clearly showed the presence of 3-CMC in the investigated evidence. After performing the analogous analysis on the urine sample, the presence of 3-CMC was also confirmed. Moreover, in the urine sample tested, the parent substance was present in trace amounts, and the dihydro metabolite was mainly present then. The metabolite corresponded to the two peaks in the chromatogram, which came from its two diastereoisomers. Since dihydro-3-CMC has two stereogenic centers, it, therefore, exists in the form of two enantiomers and two diastereoisomers. Diastereoisomers, unlike enantiomers, show differences in their physical properties, so two peaks can be observed. Differentiation of the enantiomers would require the use of an enantioselective column, where an adequate peak separation could be achieved. The liquid from the Venflon contained a trace amount of blood, which may explain the presence on the chromatogram of small peaks originating from the dihydro metabolite in the evidence tested (Figure 1).

**Figure 2.**
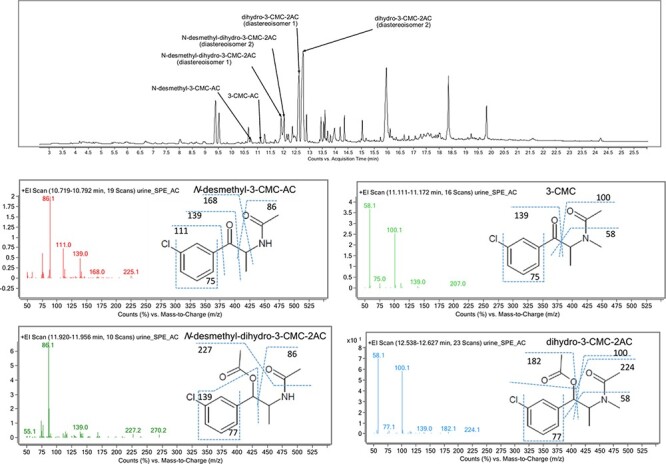
Chromatogram showing the identified acetyl derivatives of 3-CMC and its metabolites and the mass spectra of each compound according to the order of appearance on the chromatogram.

**Figure 3.**
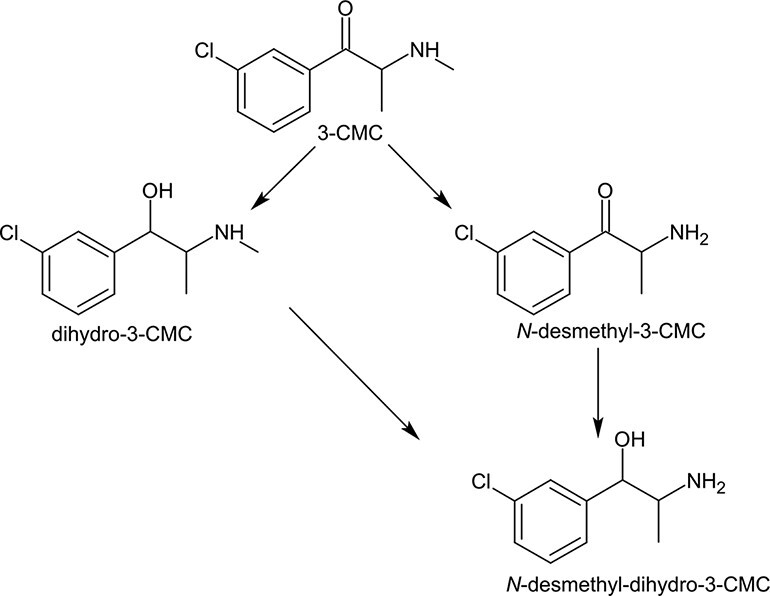
The proposed metabolism of 3-CMC.

**Figure 4.**
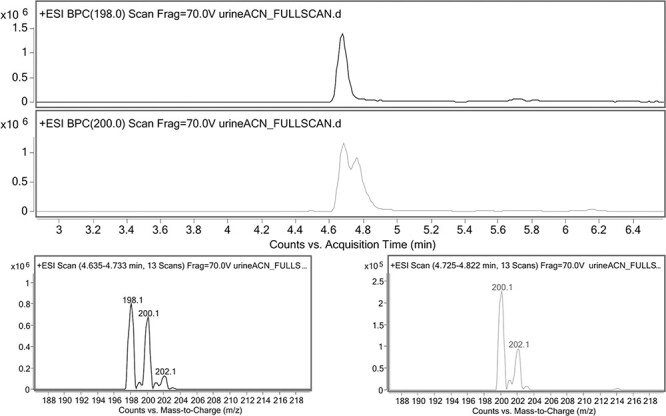
HPLC--ESI-MS chromatograms and mass spectra of 3-CMC and dihydro metabolite.

In the performed stability experiment, the parent substance was analyzed by HPLC--ESI-MS-MS, and its potential intake biomarker, the dihydro metabolite, was analyzed by HPLC--ESI-MS using the total ion current scanning option. This metabolite appears in the form of a characteristic double peak because, as noted earlier, it exists in the form of two diastereoisomers. The mass spectrum shows a pattern of isotopic peaks that is the characteristic for the presence of a chlorine atom (disturbed in the case of the parent substance due to the coelution of the dihydro metabolite), as well as the mass of the pseudomolecular ion differing by two units from the mass of the pseudomolecular ion of the parent compound (Figure 4).

The GC--MS analysis of the urine sample after acetylation showed the presence of 3-CMC as well as its metabolites *N*-desmethyl-3-CMC-AC and *N*-desmethyl-dihydro-3-CMC-2AC (two diastereoisomers), whereas mainly the dihydro-3-CMC-2AC metabolite (two diastereoisomers) was present. The metabolites were identified by comparing the obtained mass spectra with the published mass spectra of other halogen derivatives of SC (12). Details along with the characteristic molecular fragments of 3-CMC and its metabolites that were obtained using the “MassSpec Scissors” command from a free software for drawing chemical structures (ChemSketch, ACD/Labs) are shown in Figure 2. Based on the results obtained from the metabolite analysis, Figure 3 shows the proposed metabolism of 3-CMC. The metabolite dihydro-3-CMC was selected as a potential intake biomarker of 3-CMC, and a stability experiment was conducted for this metabolite as well.

**Figure 5.**
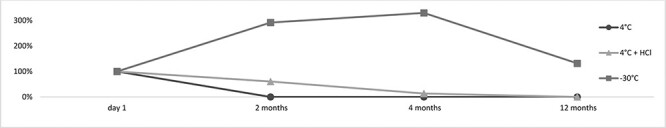
The stability of 3-CMC in the blood sample (day 1—the extraction of the biological material stored at 4°C for 24 hours).

**Figure 6.**
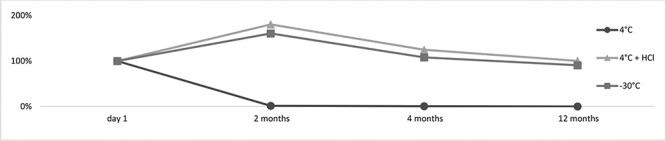
The stability of 3-CMC in the urine sample (day 1—the extraction of the biological material stored at 4°C for 24 hours).

In the stability experiments, the point of reference was the biological material, which was stored at 4°C after the autopsy. Its extraction was performed the day after the autopsy and the preservation of the biological material. This was due to the limited amount of biological material that was secured, as we were unable to determine how many time points could be determined at the beginning of the experiment. Thus, in some cases as shown in Figures 5 and 6, where the conditions, such as acidification and/or freezing, helped the stability of the test substance, a significant increase in the amount of the tested analyte can be observed. This demonstrates, therefore, how unstable the examined 3-CMC is and how significant decomposition of the substance occurs after just 24 hours of storage at 4°C. In the case of the urine sample, this increase is not as intensive as in the case of the blood sample. As such, it is clear that pH = 6.0 of the urine sample slightly protects the parent substance when compared to the blood sample.

**Figure 7.**
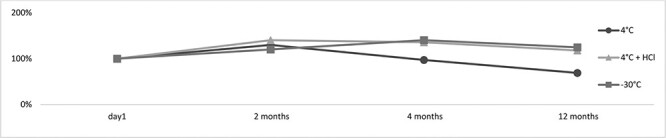
The stability of dihydro-3-CMC in the blood sample (day 1—the extraction of the biological material stored at 4°C for 24 hours).

When the blood sample was stored at ∼4°C, 3-CMC was no longer detected after 2 months. Under the same temperature conditions, in an acidified blood sample, it was possible to detect the parent substance for 4 months. At −30°C, it was possible to detect it even after 12 months. In the graph for storage temperature at −30°C (Figure 5), it can be observed that after 12 months, under these conditions, there is a decrease in the concentration similar to that observed after storage at 4°C for 24 hours.

In the urine sample that had a pH of ∼6, it was also impossible to detect 3-CMC after 2 months. After acidification and freezing, the substance was stable throughout the investigated time period (Figure 6).

**Figure 8.**
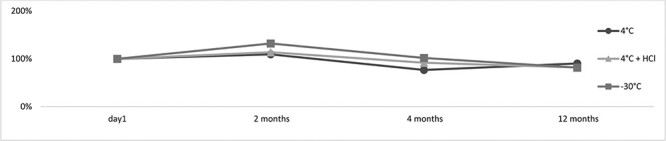
The stability of dihydro-3-CMC in the urine sample (day 1—the extraction of the biological material stored at 4°C for 24 hours).

The metabolite dihydro-3-CMC, on the other hand, showed high stability under any given conditions for 12 months in both blood and urine samples (Figures 7 and 8). Accordingly, it represents a relatively potential intake biomarker of 3-CMC. Unfortunately, the lack of commercially available standards for 3-CMC metabolites prevented the authors from quantifying dihydro-3-CMC.

While comparing the obtained concentrations of 3-CMC in the man’s blood depending on the time elapsed after the autopsy (Table III), it can be seen that these concentrations will significantly differ depending on the storage conditions. Moreover, the autopsy was conducted 2 days after the body was found. Thus, the initial concentrations of 3-CMC could have been entirely different. The concentration increase from 880 ng/mL to >2,000 ng/mL, when stored at −30°C, is a result of the short half-life of 3-CMC. When the material was frozen immediately, we obtained a high stability of 3-CMC, while the analyte had already decomposed significantly when it was stored for 24 hours at 4°C before the determination.

In the present case, toxicological tests were ordered to the Toxycology Laboratory at the same time as the shipment of physical evidence, in the form of string bags with traces of white powder (analysis result—3/4-CMC). This happened 2 months after the autopsy was performed. Actually, if the blood sample was stored at 4°C at the time the toxicological examination was ordered, the result of the analysis for the presence of the parent compound would be negative. Thus, the recommended solution is to freeze the biological material if there is any suspicion of poisoning due to NPSs, especially SC. Unfortunately, this type of information is often received very late.

**Table III.** 3-CMC Concentrations in Blood Samples Depending on Storage Conditions and Time Elapsed Since Autopsy

	3-CMC concentration in blood sample [ng/mL]		
	Storage conditions		
Time elapsed since the autopsy was performed	4°C	4°C + HCl	−30°C
1 day	880	–^a^	–^a^
2 months	0	580	2,200
4 months	0	150	2,500
12 months	0	0	1,100

a The reference point was the extract at 4°C.

The results obtained in the present study also support the suggestions made in a letter to the editor by Gerostamoulos et al. (13). The authors point out that in the case of substances from the NPS groups, a qualitative study of these substances, preferably together with their metabolites, seems more important. Although there are some exceptions, we have limited data on the pharmacology, pharmacokinetics and pharmacodynamics of these substances. Furthermore, the lack of data on the tolerance, routes of administration, dosage and sudden withdrawal syndrome of these substances makes reliable and accurate interpretation of NPS concentrations impossible. Moreover, many poisoning case reports involve polydrug intoxications, so it would be necessary to consider the issue of interactions between them as well. Thus, the toxicologist cannot comment much on the presence of NPSs beyond confirming or not confirming its occurrence. In addition, there is the stability parameter discussed in this paper and the need for studies of NPS metabolism to provide intake biomarkers of NPSs. However, the quantitative determinations of NPSs are still a very valuable source of information because knowing the reference concentration values allows us to understand NPSs and learn about the nature of these substances.

The problem of SC stability in the biological material is already rather well understood. It is influenced by a number of factors, such as the type of biological matrix, sample storage temperature, storage duration, the pH of the sample, the type of stabilizing additives used and the chemical structure of the analyte to be determined (5).

According to the published research data, in general, these substances are more stable in urine samples than in blood samples (14–16). This was also confirmed by the experiment conducted in the present study. However, there was no correlation between the concentration of SCs and their stability (15, 16). Moreover, the higher the storage temperature of the samples, the faster the degradation process occurs. SC halogen derivatives are highly sensitive to storage temperature (14–16). Thus, for blood samples containing 4-CMC, which is the constitutional isomer of 3-CMC, the estimated time for the complete degradation of this substance when stored at −26°C was 11.6 months, at 5°C ∼4 months and at room temperature 22 days. In the case of the urine sample, the time was >3 years at −26°C, 4.4 months at 5°C and 24 days at 24°C. The half-life of 4-CMC in the blood sample stored at 5°C was estimated to be 1 day, and in the sample of urine with pH = 5, it was estimated to be ∼2.5 months (14).

The stability of SCs is strongly dependent on the pH of the sample. As Sørensen (17) showed, the stability of SCs decreases significantly for pH >5.5. For pH in the range of 2.5–3.5, no significant degradation of analytes was observed after 7 days of storage at 20°C. This may explain the increased stability for urine samples. After all, in the case of urine sample, the pH can be in the range of 4.5–8. Thus, in the case of acidic urine samples, the substances will be relatively stable. However, as seen in the experiment by Adamowicz and Malczyk (14), the pH of the urine sample can change over time depending on the storage conditions of the sample. When stored at 5°C, the pH of the urine sample increased from 5.0 to 7 after 6 months, and at 24°C, it increased to  8. Adding an antimicrobial agent, such as sodium azide, to the urine sample can prevent the growth of bacteria and the subsequent alkalization of the sample.

As an additive in the role of anticoagulant to stabilize blood samples, both in clinical and forensic practice, the usual agents used are ethylenediaminetetraacetic acid (EDTA) salts, heparin, citrate buffer (citrate) and KOx. Some anticoagulants are often combined with a preservative, such as NaF, to inhibit blood glucose glycolysis by affecting enolase. In addition, NaF can inhibit the activity of many other enzymes, such as urease and cholinesterase, as well as bacterial proliferation, thereby reducing the loss of nitroaromatic compounds. The addition of NaF to various biological samples (e.g., urine, bile and vitreous humor) is a common practice in forensic medicine as it helps protect various xenobiotics from degradation (5).

However, as Sørensen (17) showed in his study, the stability is affected not only by the addition of stabilizing substances but also by their type. When a blood sample fortified with SCs was stored at room temperature with citrate as anticoagulant, the increased stability of these substances was observed when compared to the use of KOx as an anticoagulant. This is also most likely related to the pH that the reagent provides, and in the case of the sample protected by the addition of citrate, the pH was ∼5.9, and with KOx, the pH was 7.4.

Busardò et al. (18) studied the stability of mephedrone in blood samples collected intravenously and postmortem, under conditions without stabilizing additives, and with the use of EDTA as a stabilizer and also KOx with NaF. Greater stability was acquired for ante-mortem blood samples, and greater stability was obtained when KOx with NaF was used rather than EDTA as a stabilizer. The increased stability of SCs was also observed when antioxidants such as l-ascorbic acid and sodium sulfite were added to the sample (19).

The relationship between the chemical structure and the stability depends mostly on the amine’s order. The least stable are secondary amines, especially those containing a halogen atom in the aromatic ring. Tertiary amines are the most stable (15, 16). Their high stability is probably due to the fact that tertiary amines will not undergo oxidative deamination. During this process, SCs—mephedrone degradation products, known from the literature, were formed (19). An additional stabilizing effect of tertiary amines occurred when a methylenedioxy substituent was present in the aromatic ring (15, 16).

Interestingly, the studies conducted indicate that the stability is also strongly influenced by the position of the halogen substituent in the aromatic ring. For fluoromethcathinone, it was shown that the substitution at position 2 (*ortho*) of the aromatic ring is the least stable, the substitution at position 3 (*meta*) is more stable and the substitution at position 4 (*para*) is the most stable.

The most common studies in the literature are those on the stability of the parent compound. In cases where it is not possible to detect the xenobiotic, it seems important to monitor compounds that are decomposition products or metabolites. These substances may differ significantly in their properties from the parent compound and therefore may exhibit much higher stability in the biological material. Very often, it is impossible to monitor them, not only due to the limited data on metabolism or degradation but also due to the lack of commercially available standards for such substances. Consequently, there are few papers that describe the monitoring of degradation products or metabolites of SCs.

Soh and Elliott’s work showed that in the case of 4-MEC, the dihydro-4-MEC metabolite also appeared in trace amounts as a degradation product of the parent compound (20). Tsujikawa et al. (19) conducted a study on non-biological samples, in which they demonstrated probable degradation pathways of mephedrone in an alkaline environment. In addition, Maskell et al. (21) identified a degradation product of mephedrone in a formalin environment.

Czerwinska et al. (22) studied the stability of mephedrone and its five phase I metabolites, including dihydro-mephedrone, dihydro-desmethyl-mephedrone, desmethyl-mephedrone, hydroxytolyl-mephedrone and 4-carboxy-mephedrone in blood samples. Blank blood sample was protected with the addition of NaF and KOx and then stored for 10 days at 4°C and −20°C. The study showed the stability of dihydro-mephedrone and dihydro-desmethyl-mephedrone under all given conditions.

In their work, Concheiro et al. (23) demonstrated the stability of the dihydro metabolites: 4-MEC, bufedrone and mephedrone in urine samples (pH = 7.6) for 24 hours at room temperature and for 72 hours at 4°C. The same metabolites were tested in the work by Alsenedi and Morrison (24), where it was shown that these metabolites in urine samples were stable at −20°C. In contrast, they were less stable at 4°C and at room temperature, but it was still possible to detect them in samples after 201 days of storage.

In two papers, Nowak et al. (25, 26) studied the stability of 4-CMC on authentic biological material. However, they focused on evaluating the stability of the parent compound.

In a study of a serum sample, Nowak et al. (25) reported a 65% decrease in 4-CMC concentration when compared to the original extraction after just 3 days of storage at 4°C (11.5 ng/mL at day 0 to 4.0 ng/mL at day 3). In another paper, the same authors (26) studied the stability of 4-CMC in the blood sample and vitreous humor. There was a 63% decrease in the concentration in the blood sample after 30 days of storage at 4°C and after 3 days by 54% at 23°C. The blood sample stored at −15°C was stable for the entire time of the experiment (90 days).

No data have yet been found in the available literature on the stability of 3-CMC at this time. However, using the data for other halogen derivatives and the data for 4-CMC as a basis, it can be presumed that 3-CMC shows less stability than 4-CMC. There are also differences in the reported stability results for 4-CMC. The total decay time during refrigerated storage for the blank blood sample enriched with 4-CMC was ∼4 months (14). In the case of the blood sample from an authentic case, the time was 90 days (∼3 months) (26). By contrast, 113 days (∼3.7 months) after the original extraction, the concentration in the serum sample was below the LOQ, but there was a 65% decrease in concentration after just 3 days (25).

In Poland, the average time from a body’s discovery to the autopsy ranges from a few days to a few weeks (26). In our case, the autopsy was conducted 2 days after the man’s death. Autopsies are performed in our department up to 48 hours after the delivery of the corpse. Most commonly, they are conducted on the same day or the very next day. However, the biological material to the Laboratory of Toxicology can be delivered several months after the autopsy or the incident. Therefore, the storage conditions of the biological material and subsequent transport are very important.

According to the data provided by Adamowicz and Malczyk (14) compiled on the basis of nearly 3,000 samples of the biological material (mainly blood), it was estimated that the average time between the collection of the material and its delivery to the Institute of Forensic Research (IFR) in Kraków is 26 days (with a median of 10 days). A longer time was observed for material collected during the autopsy than for blood samples collected from drivers. In addition, the time from shipping the material to delivery to the IFR was 7 days (with a median of 5 days). The authors also noted a lack of control over the conditions in which the biological material is stored (usually in a refrigerator) and transported. According to the data from the literature, and the stability experiment we conducted, we found that these factors can have a significant impact on the result of SC determination. Thus, in many cases, without the monitoring of possible degradation products or metabolites, the result obtained from the determination may not reflect the concentration of SCs at the time of the event or death and will most often be falsely negative.

## Conclusion

SCs, especially their halogen derivatives like 3-CMC, demonstrate low stability in the biological material. Stability studies of 3-CMC have shown that acidification of the biological material or storage at low temperatures sustains its stability. It is crucial to monitor the presence of dihydro-3-CMC in the biological material during analysis, which showed high stability under all set storage conditions. SCs may not be detected in the biological material, and if they are, the determined concentrations at the time of testing might not correspond to the actual concentrations at the time of the event or death. Consequently, the interpretation of the results obtained for 3-CMC and dihydro-3-CMC in terms of assessing their toxicity and possible cause of death is difficult. The area of research into the search for other intake biomarkers of unstable halogenated SC derivatives remains open.

## Data Availability

The data underlying this article are available in the article.
